# Biospecimen Long-Chain N-3 PUFA and Risk of Colorectal Cancer: A Meta-Analysis of Data from 60,627 Individuals

**DOI:** 10.1371/journal.pone.0110574

**Published:** 2014-11-06

**Authors:** Bo Yang, Feng-Lei Wang, Xiao-Li Ren, Duo Li

**Affiliations:** 1 Department of Food Science and Nutrition, Zhejiang University, Hangzhou, China; 2 Department of Preventive Medicine, Wenzhou Medical University, Wenzhou, China; 3 Medical Laboratory Animal Center, Wenzhou Medical University, Wenzhou, China; IRCCS Istituto Oncologico Giovanni Paolo II, Italy

## Abstract

**Background:**

Several prospective cohort and case-control studies reported the inconsistent association between biospecimen composition of C20 and C22 long-chain (LC) n-3 polyunsaturated fatty acid (PUFA) and colorectal cancer (CRC) risk. The aim of the present study was to investigate the association of biospecimen LC n-3 PUFA with CRC risk based on prospective cohort and case-control studies.

**Methods and Results:**

Cochrane Library, PubMed, and EMBASE database were searched up to February 2014 for eligible studies. Risk ratios (RRs) or odds ratios (ORs) from prospective and case-control studies were combined using a random-effects model in the highest vs. lowest categorical analysis. Nonlinear dose-response relationships were assessed using restricted cubic spline regression models. Difference in tissue composition of LC n-3 PUFA between cases and noncases was analyzed as standardized mean difference (SMD). Three prospective cohort studies and 8 case-control studies were included in the present study, comprising 60,627 participants (1,499 CRC cases and 59,128 noncases). Higher biospecimen LC n-3 PUFA was significantly associated with a lower risk of CRC in case-control (pooled OR: 0.76; 95% CI: 0.59, 0.97; *I^2^* = 10.00%) and prospective cohort studies (pooled RR: 0.70; 95% CI: 0.55, 0.88; *I^2^* = 0.00%), respectively. A significant dose-response association was found of biospecimen C20:5n-3 (*P* for nonlinearity  = 0.02) and C22:6n-3 (*P* for trend  = 0.01) with CRC risk, respectively. Subjects without CRC have significantly higher biospecimen compositions of C20:5n-3 (SMD: 0.27; 95%: 0.13, 0.41), C22:6n-3 (SMD: 0.23; 95%: 0.11, 0.34) and total LC n-3 PUFA (SMD: 0.22; 95% CI: 0.07, 0.37) compared with those with CRC.

**Conclusions:**

The present evidence suggests human tissue compositions of LC n-3 PUFA may be an independent predictive factor for CRC risk, especially C20:5n-3 and C22:6n-3. This needs to be confirmed with more large-scale prospective cohort studies.

## Introduction

Colorectal cancer (CRC) is the most frequently diagnosed, and has a higher incidence or mortality in both women and men in developed countries than in developing countries [1]–[3]. Dietary factors were postulated to play an important role in the prevention of CRC [4]. Data from human studies suggested that dietary fatty acids, as subtypes of fat in most foods, were closely associated with the development of CRC [5], [6]. Recently, a meta-analysis of 13 prospective cohort studies [7] assessed the impact of total dietary fat on the risk of CRC, and indicated that dietary polyunsaturated fatty acid (PUFA) was not associated with the increased risk of CRC. One of the explains was that the true associations might be modified by the different effects of PUFA (omega-3 and omega-6) on the development of CRC. Seafood-derived long-chain (LC) omega-3 polyunsaturated fatty acid (n-3 PUFA), including C20:5n-3, C22:5n-3 and C22:6n-3, is suggested to reduce the risk of CRC in many epidemiological studies [8], [9]. However, dietary data from meta-analyses of prospective cohort studies provided an insufficient evidence of protective effects of dietary LC n-3 PUFA on CRC risk [10], [11], which may be due to inaccuracy in dietary assessment and an insufficient amount or variety of intake. Taking into account the difficulty in measuring dietary fatty acids accurately, more comprehensive attentions should be paid to a biomarker as a helpful tool that has been used to reflect intake closely to act as objective indices of true dietary intake. The most common biomarkers for dietary intake of LC n-3 PUFA from marine food or fish oil are C20:5n-3 and C22:6n-3, which can be determined in a variety of human biospecimens such as blood (serum/plasma/erythrocytes), adipose tissue (AT) and hair.

Accumulating evidences from in vitro and in vivo studies [12]–[14] indicate that LC n-3 PUFA as constituents of membrane phospholipids can work through several actions to protect against the initiation and early stages of CRC, including activating protein kinase C, enhancing CRC cell apoptosis, reducing inflammation and decreasing fecal bile acids as well as neutral sterol excretion. Nevertheless, results from prospective and case-control studies revealed inconsistent associations of human tissue LC n-3 PUFA with CRC risk. Most of case-control studies [15]–[17] reported that tissue composition of LC n-3 PUFA was inversely associated with CRC risk, whereas prospective cohort studies showed inverse [18] or null associations [19], [20] between tissue LC n-3 PUFA and CRC risk. Tissue compositions of LC n-3 PUFA were reported to be significantly lower in subjects with CRC (cases) compared with control subjects without CRC (noncases) in some case-control studies [21], [22], whereas inconsistent results were reported in other case-control studies [16], [17], [23]–[25].

The aim of the present study was to examine the relationship between LC n-3 PUFA compositions in human biospecimens and CRC risk based on prospective cohort and case-control studies. Additionally, the differences in biospecimens (plasma/serum/erythrocytes/whole blood/AT) compositions of LC n-3 PUFA between cases and noncases were also investigated based on case-control studies. We therefore conducted a meta-analysis to clarify the role of tissue compositions of LC n-3 PUFA in the etiology of CRC.

## Methods

### Literature research

We identified prospective and case-control studies which reported the association between LC n-3 PUFA composition in biospecimen and CRC risk from PubMed, EMBASE and Cochrane Library database up to February 2014. Search strategy was (“Fatty Acids, omega-3” AND “Colorectal Neoplasms”) for PubMed, (“Colorectal tumor” AND “omega 3 fatty acid”) for EMBASE and (“Fatty Acids, Omega-3” AND “Colorectal Neoplasms”) for Cochrane Library databases. We also searched systematic reviews from the above-mentioned database, and check the reference lists to identify studies that might have been missed. We followed MOOSE guidelines of observational studies [26] for conducting and reporting meta-analyses (Checklist S1).

### Eligibility criteria

To examine the associations of human biospecimen LC n-3 PUFA with risk of CRC, the inclusion criteria were: 1) Participants: Any aged adults from the same population; 2) Exposure: LC n-3 PUFA compositions in human biospecimen (serum/plasma/whole blood/erythrocytes/AT); 3) Outcomes: evaluating CRC incidence as outcome variable and providing risk ratio (RR) or odds ratio (OR) with the corresponding 95% confidence interval (CI) of CRC for all categories of LC n-3 PUFA compositions; 4) Study design: prospective studies (cohort, nested case-control and case-cohort study) and case-control study.

To investigate the differences in human biospecimens LC n-3 PUFA compositions between cases and noncases, the inclusion criteria were: 1) Participants: both the cases and noncases in each study were from the same population; 2) Outcomes: human biospecimen compositions of C22:6n-3, C22:5n-3, C20:5n-3 or total LC n-3 PUFA in cases and noncases; 3) Study design: case-control study.

### Definition of exposure

In the present meta-analysis, biospecimen LC n-3 PUFA composition was defined as the sum of C22:6n-3, C22:5n-3, C20:5n-3 compositions in human biospecimens (serum/plasma/whole blood/erythrocytes/AT). Blood LC n-3 PUFA composition was defined as the sum of C22:6n-3, C22:5n-3, C20:5n-3 compositions in human blood (plasma/serum/erythrocytes/whole blood).

### Data extraction

Data extraction was finished independently and performed twice by two reviewers (XLR and FLW), and disagreements were reconciled by consensus. The following data were extracted form each original study: participant characteristics (e.g., nationality, age, gender and number of participants), biospecimen LC n-3 composition as exposure of interest (e.g., measurement method, exposure source, and exposure range), biospecimen compositions of C22:6n-3, C22:5n-3, C20:5n-3 and total LC n-3 PUFA in cases and noncases, adjusted covariates and RR (OR) including 95% CI for all categories of LC n-3 PUFA composition. Our search was restricted to human studies, and we did not contact authors for the detailed information of primary studies and unpublished studies.

### Statistic analysis

We conducted two types of meta-analysis. Firstly, we performed a meta-analysis for the highest category vs. lowest. Multivariate adjusted RR (OR) for the highest vs lowest category to assess the association of biospecimen LC n-3 with CRC risk from each original study was firstly transformed to their logarithm (logRR or logOR), and the corresponding 95% CIs were used to calculate corresponding standard errors (selogRR or selogOR). Summary RR (SRR) including corresponding 95% CI as the summary risk estimate for all prospective and case-control studies was estimated using a random-effects model [27], which considers both within-study and between-study variability. The second meta-analysis was that the differences in biospecimen compositions of C22:6n-3, C22:5n-3, C20:5n-3 and total LC n-3 compositions between cases and noncases were also analyzed as standardized mean difference (SMD) by pooling the data from case-control studies, respectively. Heterogeneity among studies was assessed with the Q test and *I* ^2^ statistic. *I* ^2^ statistic describes the proportion of total variation attributable to between-study heterogeneity as opposed to random error or chance. We defined the low, moderate and high degrees of heterogeneity by *I* ^2^ values of 25%, 50% and 75% as cut-off points [28], and considered an *I* ^2^ value greater than 50% as indicative of heterogeneity according to Cochrane Handbook. In the presence of substantial heterogeneity, stratified analysis was conducted to identify the possible sources of heterogeneity by study design (case-control and prospective study), different regions (Asia and West (Europe and USA)), gender (women and men) and biospecimen types (blood and adipose tissue). Meta-regression with restricted maximum likelihood (REML) estimation was conducted to assess the potentially important covariates exerting substantial impact on between-study heterogeneity. Sensitivity analysis was performed to evaluate possible influence of individual study with potential bias on overall risk. Publication bias was quantitatively examined by Begg's test and Egger's regression test [29].

Furthermore, the dose-response association of biospecimen LC n-3 PUFA with CRC risk was performed in the present study. Original studies with 3 or more categories were included in the dose-response analysis. Midpoint of upper and lower boundaries was taken as the dose of the quantile if the study only reported the range; if the highest quantile was open-ended, its dose was regarded as 1.2-fold the highest boundary [30]; if the lowest quantile or reference category was open-ended, the midpoint of lowest boundary and zero was taken as the dose of lowest quantile. A nonlinear (curvilinear) trend was tested by using a 2-stage random-effects dose-response meta-analysis [31], [32]. The compositionof LC n-3 PUFA in biospecimen was modeled by using restricted cubic splines with 3 knots (2 spline transformations) at percentiles (25%, 50%, and 75%) of the distribution [33]. A *P*-value for nonlinearity was calculated by testing the null hypothesis that the coefficient of the second spline is equal to zero [34]. In the presence of substantial linear trends (*P*-value for nonlinearity >0.05), a linear dose-response analysis [32] was conducted to examine the association between every 1% increment of LC n-3 PUFA composition in biospecimens and the risk of CRC. Statistical analyses of the combined data were performed by STATA version 11.0 (Stata CORP, College Station, TX). *P*-value less than 0.05 were considered statistically significant in the present study.

## Results

### Study characteristics

**Figure 1:**
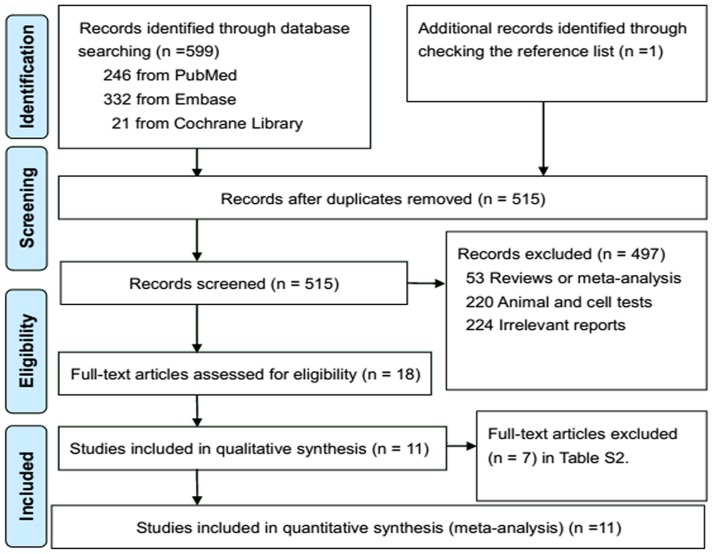
PRISMA Flow Diagram for included prospective cohort and case-control studies.

We identified 599 potential studies from electronic search, and 515 studies were left after removing duplicates. Eleven relevant studies were eligible for the present study after full text review (Figure 1; Table S1). A total of 1,499 CRC cases among 60,627 participants were included in the present study, separately from 3 prospective cohort [18]–[20] and 8 case-control studies [15]–[17], [21]–[25]. Of the 3 cohort studies, 2 studies were conducted in West (Europe [19] and USA [20]), and 1 study was conducted in Japan [18]. Of the 8 case-control studies, 5 studies were conducted in Europe [15], [16], [22], [24], [25], and 3 studies were conducted in Japan [17], [21], [23]. Of the 11 included studies, 6 studies assessed serum (plasma) biomarker of LC n-3 composition [15], [18], [20], [21], [23]–[25], 4 studies assessed RBC biomarker [17], [19], [21], [24], 1 study [20] assessed whole blood biomarker and 3 studies assessed AT biomarker [16], [21], . LC n-3 PUFA compositions in biospecimens were quantified by gas liquid chromatography (GLC), and measurement unit was percentage of total fatty acids (% tFC) except for 1 study (mg/dL) [23]. Two studies separately provided data of males (M) and females (F) [18], [23], 1 study only provided data of females [19], and 1 study only provided data of males [20]. For the analysis on association between biospecimen LC n-3 PUFA and CRC risk, the characteristics of included 3 prospective cohort and 4 case-control studies were summarized in Table 1. For the analysis of different biospecimen LC n-3 PUFA compositions between subjects with CRC (cases) and control subjects without CRC (noncases), the characteristics of included 8 case-control studies were summarized in Table 2.

**Table 1:** Characteristics of included prospective and case-control studies in the meta-analysis of association between biospecimen long-chain n-3 PUFA and the risk of colorectal cancer.

Author (year)	Design (Nation)	Population (case/participants)	Gender (Age)	Exposure of interest		Outcomes RR/OR (95%CI)	Covariates adjusted
				Measurement	Range (H vs. L)		
Cottet (2013)	Cohort (France)	Subjects from E3N-EPIC cohort (328/19934)	F 40–65 y	Erythrocyte GLC (% tFC)	EPA:>1.18 vs. <0.89	0.88 (0.62–1.25)	BMI, physical activity, energy intake, alcohol consumption, smoking, educational level, menopausal status, and family history
					DHA:>7.21 vs. <6.10	0.83 (0.57–1.22)	
							
Pot (2008)	Case-control (Netherlands)	Subjects from the POLIEP study (498/861)	Both 18–75 y	Serum GLC (% tFC)	EPA:>1.00 vs. <0.70	0.79 (0.55–1.15)	Family history, BMI, indication for endoscopy, physical activity, smoking regular use of drugs, hormone replacement therapy, diet change due to gastrointestinal complaints, and daily intake of energy, alcohol, fat, fiber, red meat, vegetables and legumes and cholesterol.
					DHA:>0.60 vs. <0.50	0.71 (0.49–1.02)	
							
Ghadimi (2008)	Case-control (Japan)	Subjects form study on dietary, lifestyle factors and colon cancer (203/382)	F and M 35–75 y	Serum GLC (% tFC)	**F**:		Age, BMI, history of CRC, history of diabetes; smoking, alcohol consumption, vigorous exercise and season of data collection.
					EPA:>10.48 vs. <5.50	0.62 (0.22–1.80)	
					DPA:>1.80 vs. <1.00	1.60 (0.35–7.31)	
					DHA:>20.2 vs. <13.8	0.43 (0.14–1.38)	
					LC n-3:>32.8 vs. <21.3	0.54 (0.19–1.54)	
					**M**:		
					EPA:>9.50 vs. <5.70	1.22 (0.48–3.54)	
					DPA:>1.70 vs. <1.10	0.40 (0.15–1.09)	
					DHA:>19.2 vs. <14.2	2.30 (0.675–7.35)	
					LC n-3:>29.5 vs. <21.8	0.86 (0.28–2.21)	
Hall (2007)	Cohort (USA)	Subjects from the primary prevention of cancer and CVD disease (178/14916)	M 40–84 y	Whole bloodGLC (% tFC)	EPA: 2.21–4.07 vs. 0.89–1.55	0.60 (0.29–1.23)	BMI, multivitamin use, history of diabetes, useof drugs, vigorous exercise, alcohol intake, and quartile of red meat intake.
					DHA: 2.83–6.22 vs. 0.69–1.87	0.69 (0.39–1.23)	
					LC n-3: 6.06–11.41 vs. 2.43–4.43	0.60 (0.32–1.11)	
Kuriki (2006)	Case-control (Japan)	Subjects form Hospital-based Epidemiologic Research Program; (74/295)	Both 20–80 y	EM; GLC (% tFC)	EPA:>1.70 vs. <1.18	0.69 (0.32–1.50)	BMI, habitual exercise, drinking and smoking status, green-yellow vegetable intake, and family history.
					DPA:>1.50 vs. <1.24	0.83 (0.34–2.05)	
					DHA:>6.10 vs. <5.06	0.36 (0.14–0.93)	
Kojima (2005)	Cohort (Japan)	Subjects from Collaborative Cohort Study for the Evaluation of Cancer Risk (JACC Study); 169/23863;	F and M 20–81y	Serum; GLC (% tFC)	**F**:		Family history, BMI, education, smoking and alcohol drinking, green leafy vegetable intake, and physical exercise
					F-EPA:>3.33 vs. <1.73	0.83 (0.39–1.80)	
					DPA:>0.94 vs. <0.66	0.64 (0.30–1.39)	
					DHA:>5.92 vs. <4.20	0.80 (0.33–1.93)	
					**M**:		
					EPA:>3.84 vs. <1.91	0.44 (0.18–1.08)	
					DPA:>1.02 vs. <0.68	0.30 (0.11–0.80)	
					DHA:>6.25 vs. <4.23	0.23 (0.07–0.76)	
Busstra (2003)	Case-control (Netherlands)	Subjects from colorectal adenomas study; 52/109;	Both <75 y	Adipose; GLC (% tFC)	LC n-3:>0.27 vs. <0.20	0.30 (0.10–1.10)	Age, family background, energy intake and gender

Abbreviations: H: the highest category; L: the lowest category; F: females; M: males; GLC: Gas liquid chromatography; tFC: total fatty acid; EPA: eicosapentaenoic acid (C20: 5n-3); DPA: docosapentaenoic acid (C22: 5n-3); DHA: docosahexaenoic acid (C22: 6n-3); LC n-3: long-chain n-3 polyunsaturated fatty acid (C20: 5n-3+C22: 5n-3+C22: 6n-3).

**Table 2:** Characteristics of included case-control studies in the mea-analysis of different long-chain n-3 PUFA compositions between subjects with and without colorectal cancer.

Author (year)	Design (Nation)	Gender (F or M)	Mean age (years)	No. of subjects		Biospecimen LC n-3 PUFA compositions (% total fatty acids)			
				Cases	Controls	Biospecimen	Subtypes	Case vs. Control (Mean ±SD)	*P* a value
Okuno (2013)	Case-control (Japan)	Both	60.5	41	61	Plasma	C20:5n-3	2.27±1.14 vs 3.04±1.65	0.006
						Plasma	C22:5n-3	0.99±0.26 vs1.00±0.22	0.80
						Plasma	C22:6n-3	7.63±1.43 vs 7.84±1.59	0.49
						Erythrocyte	C20:5n-3	1.86±0.71 vs 2.23±0.85	0.02
						Erythrocyte	C22:5n-3	2.11±0.30 vs 2.12±0.27	0.93
						Erythrocyte	C22:6n-3	7.81±0.90 vs 7.90±1.06	0.64
						Adipose	C20:5n-3	0.12±0.19 vs 0.10±0.07	0.62
						Adipose	C22:5n-3	0.28±0.18 vs 0.25±0.22	0.37
						Adipose	C22:6n-3	0.58±0.32 vs 0.66±0.39	0.28
Giuliani (2013)	Case-control (Italy)	Both	60.0	52	50	Adipose	C20:5n-3	0.02 ± 0.03 vs 0.02±0.03	NS
						Adipose	C22:5n-3	0.16±0.10 vs 0.15±0.12	NS
						Adipose	C22:6n-3	0.16±0.12 vs 0.14±0.12	NS
Pot (2008)	Case-control (Netherlands)	Both	46.5	363	498	Serum	C22:5n-3	0.80±0.50 vs 0.80±0.60	NS
						Serum	C22:6n-3	0.60±0.30 vs 0.60±0.30	NS
Ghadimi (2008)	Case-control (Japan)	F	58.0	55	112	Serum	C20:5n-3	0.24±0.13 vs 0.27±0.15	0.12
						Serum	C22:5n-3	0.07±0.03 vs 0.05±0.04	0.001
						Serum	C22:6n-3	0.48±0.16 vs 0.56±0.31	0.02
						Serum	LC n-3	0.79±0.27 vs 0.88±0.54	0.18
		M	60.0	148	67	Serum	C20:5n-3	0.24±0.10 vs 0.26±0.12	0.220.
						Serum	22:5n-3	0.07±0.04 vs 0.06±0.05	001
						Serum	C22:6n-3	0.50±0.25 vs 0.57±0.20	0.06
						Serum	LC n-3	0.80±0.34 vs 0.89±0.37	0.53
Kuriki (2006)	Case-control (Japan)	Both	50.0	74	221	Erythrocyte	C20:5n-3	1.40±0.50 vs 1.50±0.60	NS
						Erythrocyte	C22:5n-3	1.40±0.40 vs 1.40±0.30	NS
						Erythrocyte	C22:6n-3	5.30±1.00 vs 5.50±1.30	NS
						Erythrocyte	LC n-3	8.10±1.60 vs 8.40±2.00	NS
Busstra (2003)	Case-control (Netherlands)	F	46.5	52	57	Adipose	LC n-3	1.10±0.09 vs 1.14±0.10	NS
Baro (1998)	Case-control (Spain)	Both	58.5	17	29	Plasma	C20:5n-3	0.96±0.19 vs 0.79±0.21	NS
						Plasma	C22:5n-3	0.70±0.05 vs 0.65±0.09	NS
						Plasma	C22:6n-3	3.02±0.18 vs 2.91±0.28	NS
						Erythrocyte	C22:6n-3	6.25±0.20 vs 6.34±0.33	NS
Fernandez-Banares (1996)	Case-control (Spain)	Both	62.0	22	12	Plasma	C20:5n-3	0.46± 0.03 vs 0.70±0.07	0.001
						Plasma	C22:5n-3	0.58± 0.06 vs 0.47±0.05	NS
						Plasma	C22:6n-3	3.42± 0.19 vs 3.45±0.15	NS

Abbreviations: F: females; M: males; NO.: Number; NS: no significance; LC n-3: long-chain n-3 polyunsaturated fatty acid (C20: 5n-3+C22: 5n-3+C22: 6n-3).

a *P* value for different LC n-3 PUFA compositions between cases and controls.

### Highest vs lowest category

**Figure 2:**
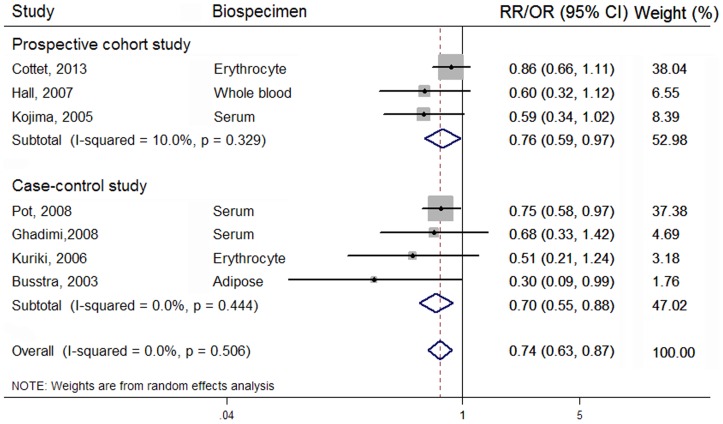
Forest plot corresponding to the random-effects meta-analysis quantifying the relationship between LC n-3 PUFA composition and CRC risk for the highest vs. lowest category. Relative risks (RRs) or odds ratios (ORs) compared the highest vs. lowest category of biospecimen LC n-3 PUFA composition and were grouped by study designs. The size of the gray box representing each risk estimate was proportional to the weight that the risk estimate contributed to the summary risk estimate. The diamonds denoted summary risk estimate.

Overall, 3 prospective cohort [18]–[20] and 4 case-control studies [15]–[17], [23] were eligible for the meta-analysis on the association of LC n-3 PUFA composition in biospecimens with CRC risk, comprising 60,360 participants. A significantly inverse association between biospecimens LC n-3 PUFA and CRC risk was observed in 60,360 participants (summary RR = 0.74; 95% CI: 0.63, 0.87), with no between-study heterogeneity (*I^2^* = 0.00%) (Figure 2). Biospecimen C20:5n-3 and C22:6n-3 were both inversely associated with the risk of CRC, and the summary RR was 0.78 (95% CI: 0.64, 0.96; *I* ^2^ = 0.00%) and 0.68 (95% CI: 0.54, 0.84; *I* ^2^ = 0.00%), respectively (Table 3). However, no significant association was found of biospecimen C22:5n-3 with CRC risk in 15,593 participants from 1 prospective and case-control studies (summary OR = 0.80; 95% CI: 0.42, 1.52; *I* ^2^ = 47.60%).

**Table 3:** Subgroup analysis for association between biospecimen long-chain n-3 PUFA and risk of colorectal cancer.

Factors stratified	C20:5n-3					C22:6n-3					LC n-3 PUFA				
	*N* a	SRR (95% CI)	Heterogeneity		*P* c	*N*	SRR (95% CI)	Heterogeneity		*P*	*N*	SRR (95% CI)	Heterogeneity		*P*
			I^2^ (%)	*P* b				I^2^ (%)	*P*				I^2^ (%)	*P*	
Overall analysis	6	0.78 (0.64, 0.96)	0.00	0.89		6	0.68 (0.54, 0.84)	0.00	0.42		7	0.74 (0.63, 0.87)	0.00	0.50	
Study design					0.93					0.37					0.63
PC	3	0.77 (0.58, 1.00)	0.00	0.49		3	0.76 (0.56, 1.01)	0.00	0.30		3	0.76 (0.59, 0.97)	10.00	0.33	
CC	3	0.79 (0.58, 1.05)	0.00	0.89		3	0.55 (0.36, 0.85)	30.60	0.00		4	0.70 (0.55, 0.88)	0.00	0.45	
Regions					0.65					0.10					0.30
West (USA and Europe)	4	0.81 (0.63, 1.02)	0.00	0.64		3	0.75 (0.59, 0.96)	0.00	0.80		4	0.76 (0.61, 0.93)	19.70	0.29	
Asia	2	0.72 (0.48, 1.07)	0.00	0.77		3	0.34 (0.13, 0.85)	43.00	0.17		3	0.60 (0.40, 0.89)	0.00	0.88	
Gender					0.42					0.24					0.14
Female	3	0.85 (0.62, 1.15)	0.00	0.82		2	0.78 (0.56, 1.09)	0.00	0.56		3	0.83 (0.66, 1.05)	0.00	0.70	
Male	3	0.65 (0.19,1.11)	13.00	0.32		5	0.47 (0.26, 0.88)	33.00	0.22		3	0.53 (0.33, 0.84)	13.70	0.32	
Biospecimens					0.68					0.58					0.73
Serum	3	0.77 (0.57, 1.02)	0.00	0.57		3	0.63 (0.45, 0.86)	0.00	0.38		3	0.71 (0.57, 0.89)	0.00	0.73	
Erythrocytes	2	0.80 (0.60, 1.06)	0.00	0.74		2	0.70 (0.48, 1.01)	23.50	0.27		2	0.79 (0.55, 1.15)	17.80	0.27	
Whole blood	1	0.80 (0.60, 1.06)	.	.		1	0.69 (0.39, 1.23)	.	.		1	0.60 (0.32, 1.12)	.	.	

Abbreviations: LC n-3: long-chain n-3 polyunsaturated fatty acid (C20: 5n-3+C22: 5n-3+C22: 6n-3); SRR: summary risk ratio; PC: prospective cohort study; CC: case-control study.

a *N*, number of included studies.

b *P* value for heterogeneity within subgroup.

c *P* value for heterogeneity between subgroups with meta-regression analysis.

In stratified analysis (Table 3), there was no evidence that the estimated summary RR differed significantly by sex (*P* for meta-regression  = 0.14). We further focused on the difference in pooled association estimate between prospective cohort and case-control study. In 3 cohort studies, biospecimen C20:5n-3, C22:6n-3 and LC n-3 PUFA was all significantly associated with the lower risk of CRC, and the pooled RR was 0.77 (95% CI: 0.58, 1.00; *I^2^* = 0.00%), 0.76 (95% CI: 0.56, 1.01; *I^2^* = 0.00%), and 0.76 (95% CI: 0.59, 0.97; *I* ^2^ = 10.00%), respectively. However, only one prospective cohort study separately reported an association between serum C22:5n-3 and CRC risk in Japan males and females [18], and the pooled RR is 0.47 (95% CI: 0.23, 0.97; *I* ^2^ = 28.60%). In 4 case-control studies, higher biospecimen LC n-3 composition was also significantly associated with a lower risk of CRC (pooled OR = 0.70; 95% CI: 0.55, 0.88; *I^2^* = 0.00%), especially biospecimen C22:6n-3 (pooled OR = 0.55; 95% CI: 0.36, 0.85; *I^2^* = 30.60%), whereas biospecimen C20:5n-3 exhibited no significant association (pooled OR = 0.79; 95% CI: 0.58, 1.05; *I^2^* = 0.00%). There were 2 case-control studies reported the association of biospecimen C22:5n-3 with CRC risk [17], , and the pooled OR is 1.25 (95% CI: 0.65, 2.41; *I^2^* = 0.00%). However, the results of the meta-regression did not show the significant difference between the two study designs. No evidence of significant difference was found between other subgroups with meta-regression (Table 3).

In a sensitivity analysis, we sequentially omitted 1 study at a time and reanalyzed the remaining data. We found that exclusion of any individual study did not substantially change the overall association. In publication bias analysis, there was also no indication of publication bias as suggested by visual inspection of Begg's funnel plot (*P* for bias  = 0.452) and Egger's regression test (*P* for bias  = 0.175).

### Difference in LC n-3 PUFA compositions between cases and noncases

**Figure 3:**
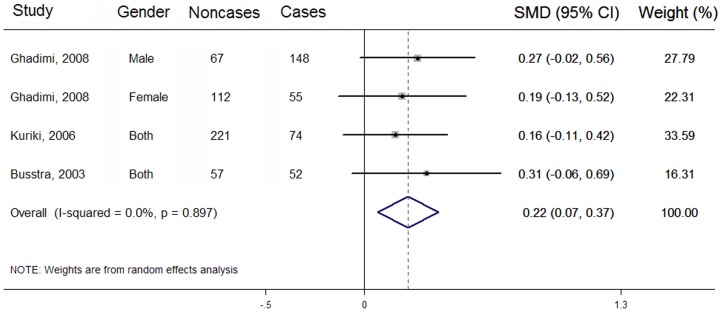
Forest plot corresponding to the random-effects meta-analysis analysis on difference in biospecimen compositions of LC n-3 PUFA between cases and noncases. Case-control studies are referred to by first author, year of publication, gender and biospecimen types. The combined standardized mean difference (SMD) was achieved using random-effects model. Grey square represents SMD in each study, with square size reflecting the study-specific weight and the 95% CI represented by horizontal bars. SMD from individual study were pooled by random effect model. The diamond indicates summary SMD.

There were 4 case-control studies eligible for the analysis of different biospecimen total LC n-3 PUFA composition between cases and noncases [15]–[17], [23]. Compared with 329 cases, 457 noncases have a significantly higher LC n-3 PUFA composition in biospecimens (SMD: 0.22; 95% CI: 0.07, 0.37), with no between-study heterogeneity (*I* ^2^ = 0.00%) (Figure 3). Similarly, there were 7 case-control studies eligible for analyses on different biospecimen C20:5n-3 and C22:6n-3 composition between cases and noncases [15], [17], [21]–[25], respectively. Compared with 623 CRC cases, 1315 noncases have significantly higher biospecimen C20:5n-3 (SMD: 0.27; 95% CI: 0.13, 0.41; *I* ^2^ = 36.20%) and C22:6n-3 composition (SMD: 0.23; 95% CI: 0.11, 0.34; *I* ^2^ = 0.00%) (Table 4). However, no significant difference was found in biospecimen C22:5n-3 composition between 587 cases and 817 noncases from 6 case-control studies (SMD: −0.08; 95% CI: −0.22, 0.06; *I* ^2^ = 17.60%) (Table 4).

**Table 4:** Subgroup analysis for different biospecimen long-chain n-3 PUFA compositions between subjects with and without CRC.

Factors stratified	C20:5n-3					C22:5n-3					C22:6n-3				
	*N* a	SMD (95% CI)	Heterogeneity		*P* c	*N*	SMD (95% CI)	Heterogeneity		*P*	*N*	SMD (95% CI)	Heterogeneity		*P*
			*I* ^2^ (%)	*P* b				*I* ^2^ (%)	*P*				*I* ^2^ (%)	*P*	
Overall analysis	7	0.27 (0.13, 0.41)	36.20	0.18		6	−0.08 (−0.22, 0.06)	17.60	0.17		7	0.23 (0.11, 0.34)	0.00	0.38	
Region					0.74					0.12					0.45
West	4	0.29 (−0.10, 0.67)	56.00	0.07		3	−0.33 (−0.64, 0.00)	0.00	0.43		4	0.30 (−0.06, 0.67)	51.00	0.08	
East	3	0.24 (0.08, 0.40)	27.90	0.23		3	−0.02 (−0.16, 0.11)	0.00	0.44		3	0.24 (0.11, 0.38)	0.00	0.94	
Gender					0.90					0.22					0.25
Female	1	0.22 (−0.10, 0.55)	.	.		1	−0.28 (−0.61, 0.04)	.	.		1	0.29 (−0.04, 0.61)	.	.	
Male	1	0.34 (0.14, 0.55)	.	.		1	0.05 (−0.15, 0.25)	.	.		1	0.37 (0.21, 0.52)	.	.	
Both	5	0.26 (0.05, 0.46)	50.10	0.05		5	−0.03 (−0.28, 0.05)	12.60	0.33		5	0.18 (0.03, 0.33)	10.80	0.34	
Biospecimens					0.05					0.54					0.55
Blood	5	0.30 (0.15, 0.44)	17.60	0.27		4	−0.04 (−0.20, 0.11)	26.70	0.12		5	0.24 (0.10, 0.40)	18.0	0.25	
Adipose	2	−0.07 (−0.35, 0.20)	0.00	0.59		2	−0.02 (−0.35, 0.30)	28.10	0.24		2	0.19 (−0.09, 0.47)	0.00	0.84	

Abbreviations: CRC, colorectal cancer; *N*: number of included case-control studies; PC: prospective cohort study; CC: case-control study; SMD: standard mean difference compared with colorectal cancer subjects.

a *N*, number of included studies.

b *P* value for heterogeneity within each subgroup.

c *P* value for heterogeneity between subgroups with meta-regression analysis.

Stratified analysis indicated that there was a significantly higher blood C20:5n-3 and C22:6n-3 compositions in noncases compared with cases, whereas adipose tissue C20:5n-3 and C22:6n-3 compositions exhibited no significant difference between cases and noncases (Table 4). Results from meta-regression only showed a significant difference in C20:5n-3 composition between the two biospecimens (*P* value  = 0.05). Furthermore, biospecimen compositions of C20:5n-3 and C22:6n-3 were both significantly higher in Asian noncases compared with cases, whereas no significant difference was observed in Western noncases compared with cases. There was no significant difference between the two populations with meta-regression (Table 4).

### Dose-response analysis

**Figure 4:**
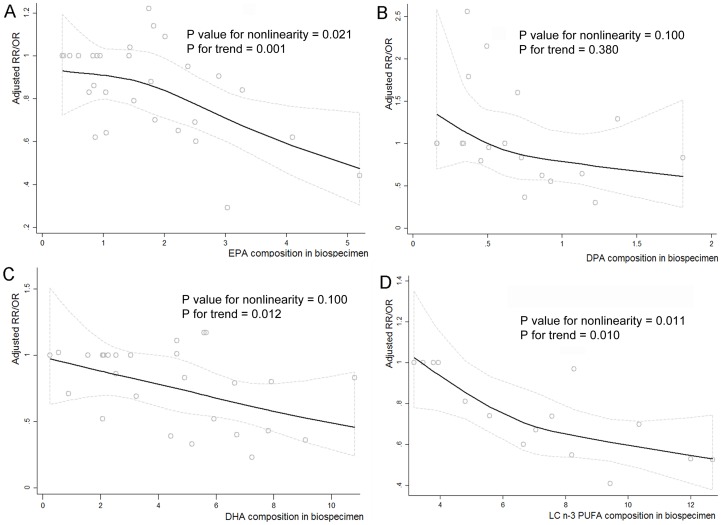
Nonlinear dose-response trend analysis assessed by restricted cubic spline model with three knots. Adjusted ORs (RRs) from all category of biospecimen C20:5n-3, C22:5n-3, C22:6n-3 and total LC n-3 PUFA in each study were separately represented by the small gray circle in the figure A, B, C and D, and corresponding nonlinear dose-response relationship was represented by the black solid line shown in figure A, B, C, and D by using restricted cubic splines functional model with three knots at percentiles 25%, 50%, and 75% of the distribution, respectively.

Four eligible studies were available to evaluate the dose-response association of biospecimen LC n-3 PUFA with CRC risk [16], [17], [20], [23], and there was a significantly nonlinear trend in 15,593 participants (*P* for nonlinearity  = 0.01; *P* for trend  = 0.01) (Figure 4D). Six eligible studies were available to evaluate the dose-response analysis on relationship of biospecimen C20:5n-3 and C22:6n-3 with CRC risk in 60,291 participants [15], [17]–[20], [23], respectively (Figure 4 A and C). A significantly nonlinear dose-response association was observed between biospecimen C20:5n-3 and CRC risk (*P* for nonlinearity  = 0.021; *P* for trend  = 0.001). The nonlinear association between biospecimen C22:6n-3 and CRC risk was not significant (*P* for nonlinearity  = 0.10), but the overall association in the linear dose-response model was significant (*P* for trend  = 0.012), with a 1% increment of biospecimen C22:6n-3 composition associated with 5% reduced risk of CRC (pooled RR = 0.95; 95% CI: 0.92, 0.98; *I^2^* = 15.00%). Likewise, three relevant studies were available to evaluate the dose-response relationship between biospecimen C22:5n-3 and CRC risk [17], [18], [23], but no significant nonlinear and linear trend was observed in 24,540 individuals, respectively (*P* for nonlinearity  = 0.10; *P* for trend  = 0.38) (Figure 4B).

## Discussion

To our knowledge, this is the first meta-analysis evaluating an association between biospecimen LC n-3 PUFA and CRC risk. Overall, our findings suggested that biospecimen composition of LC n-3 PUFA was inversely associated with CRC risk, especially C20:5n-3 and C22:6n-3. Tissue compositions of C20:5n-3, C22:6n-3 and total LC n-3 PUFA were all significantly lower in CRC subjects compared with controls without CRC.

There are several hypothesized mechanisms explaining the possible protective role of tissue LC n-3 PUFA in the etiology of CRC carcinogenesis. The lower composition of LC n-3 PUFA in cases was observed in the present study, suggesting that the characteristic LC n-3 PUFA composition in biospecimen may be involved in the mechanism of CRC progression. Firstly, LC n-3 PUFA existing in biomembrane phospholipids has been found to be involved in the regulation of downstream receptor activity, cell proliferation and apoptosis process by alternation of the fluidity, structure and/or function of lipid rafts or caveolae located in cell surface [13], [35], [36]. In addition, LC n-3 PUFA, especially C20:5n-3, can may modulate cyclooxygenases (COX) activity, leading to the reduction of n-6 family derived 2-series PG (e.g., PGE_2_) with promoting tumor growth effects [37] in favor of n-3 family derived 3-series PG (e.g., PGE_3_) with suppressive effects in several cell types including CRC cells [38], [39]. Lastly, LC n-3 PUFA may have an antineoplastic effect through alteration in the cellular redox state and increased oxidative stress. The evidence from experimental studies indicated that the oxidative stress and colonocyte apoptosis induced by the fermentation product short-chain fatty acid butyrate might be potentiated by both intrinsic and extrinsic apoptosis pathways medicated by C22:6n-3 [36], [40].

In subgroup analyses by study design for the highest category vs lowest, the pooled association estimates of LC n-3 PUFA including the specific subtypes were all significant in prospective cohort studies, although the pooled estimate of C20:5n-3 and C22:5n-3 from case-control studies did not reach statistical significance. Prospective cohort studies greatly decreased the possibility of recall bias and selection biases, which are always inherent in retrospective case-control studies, in view of exposure information was collected before suffering from the disease. Thus, prospective cohort designs are warranted to elucidate causal relationships. In addition, the difference in the number and types of the potential covariates adjusted in multivariable statistic models probably resulted in the discrepant results between cohort and case-control studies. Therefore, we cannot exclude the possibility that the true associations might be distorted in case-control studies adjusting for few confounding factors. For the stratified analysis of LC n-3 PUFA composition between cases and noncases by biospecimen types, blood (serum/plasma/erythrocytes) compositions of LC n-3 PUFA revealed that a statistically significant difference between cases and noncases, but not AT. The heterogeneity between the two biospecimens may be partially explained by different metabolic characteristics of these human biospecimens (serum/plasma/erythrocyte/AT) as biomarkers. Plasma (serum) biomarker represents a combination of triacylglycerol, cholesterol esters and phospholipids found in lipoproteins, and reflects dietary intakes of the past few hours or days [41]. Determination of LC n-3 PUFA composition in erythrocytes provides a biomarker for over several weeks, considering that the half-life of erythrocytes is 120 days [42]. Conversely, AT biomarker represents mostly triacylglycerol, and reflects long-term dietary intake of fatty acids, mainly because of its slow turnover and lack of response to acute diseases. However, given that concentrations of PUFA are very low in AT [43], this tissue does not seem to be a perfect biomarker of LC n-3 PUFA. Furthermore, although biospecimen compositions of fatty acids were mainly determined by GLC in these observational studies, all measurement steps may be accomplished by different methodological procedures, chemicals, and equipments. Hence, the difference between blood and AT biomarker of LC n-3 PUFA may be partly attributed to no agreed standard procedures.

Several strengths could be highlighted in our study. Firstly, our present study showed a characteristic biospecimen compositions of LC n-3 PUFA associated with CRC risk, and further analyzed the difference in the compositions of biospecimen LC n-3 PUFA between CRC cases and noncases. In addition, a biomarker as an indicator of some biological state or condition can closely reflect an integrated measurement of diet over time, thus recall bias might not occur in case-control studies. Finally, no evidence of potential publication bias was observed in the present study, though publication bias could be of concern because small studies with null results tend not to be published. In addition, there are also several potential limitations in the present study. Firstly, meta-analyses of observational studies are susceptible to inherent biases (e.g., report biases and unknown residual confoundings), which might have affected the summarized results. Secondly, selection bias might be unavoidable, due to inclusion of case-control studies with regards to elucidating causal relationships. Thirdly, data on association of tissue biomarker with colon or rectal cancer risk was not provided by the original studies, respectively. Given that therapeutic strategy for colon cancer and rectal cancer may differ [2], [44], future epidemiological studies should further investigate whether LC n-3 PUFA in human tissues could be beneficial for colon or rectal cancer, respectively. Fourthly, the limited numbers of cohort studies included in this meta-analysis might diminish the statistical power to detect the association between biospecimen LC n-3 PUFA and CRC risk. Therefore, care must be exercised in the extrapolation of our findings to larger populations of CRC individuals.

In conclusion, this meta-analysis provided a sufficient evidence that human biospecimen LC n-3 composition was inversely associated with the risk of CRC, especially C20:5n-3 and C22:6n-3. Populations without CRC have higher tissue compositions of C20:5n-3, C22:6n-3 and total LC n-3 PUFA than those with CRC. These evidences have important public health implications for CRC prevention. LC n-3 PUFA profile in human tissues may be an independent predictive factor for CRC risk. Future epidemiological studies should focus on whether the risk of colon or rectal cancer could be modified by increasing LC n-3 PUFA composition in human tissues, respectively. Nevertheless, the protective effect of human tissue LC n-3 PUFA on CRC risk needs to be replicated by further large-scale prospective studies.

## Supporting Information

Checklist S1 **MOOSE Checklist of the Present Meta-Analysis.**
(DOC)

Table S1 **Observational studies Excluded from Meta-Analysis.**
(DOC)
