# Supplementary material for: Biospecimen Long-Chain N-3 PUFA and Risk of Colorectal Cancer: A Meta-Analysis of Data from 60,627 Individuals
Source: PLoS One. 2014 Nov 6;9(11):e110574. doi: 10.1371/journal.pone.0110574 (PMC4222788; doi:10.1371/journal.pone.0110574)
Supplement: Checklist S1 — MOOSE Checklist of the Present Meta-Analysis. (DOC) [file pone.0110574.s001.doc]

**Checklist S1**

| **MOOSE Checklist of the Present Meta-Analysis** | | |
| --- | --- | --- |
| **Criteria** | **Comments of how the criteria were handled in the meta-analysis** | **Reported on page #** |
|  | **Reporting of background should include** |  |
| Problem definition | Epidemiological findings obtained on biospecimen long-chain (LC) n-3 PUFA associated with CRC risk are still controversial; therefore, the evidence of association between LC n-3 composition in biospecimen and CRC risk remains to be summarized quantitatively. | 3, 4 |
| Hypothesis statement | FAs composition in biological specimen as a biomarker of individual dietary intake may play a critical role in the etiology of CRC, especially n-3 polyunsaturated fatty acid (PUFA). | 4 |
| Description of study outcomes | Results of epidemiological studies are still inconsistent concerning relationship between LC n-3 PUFA composition in biospecimen and CRC risk. | 4 |
| Type of exposure or intervention used | LC n-3 PUFA composition in biospecimen (serum/plasma/RBCs/whole blood/adipose) including eicosapentaenoic acid (EPA, 20:5n-3), docosapentaenoic acid (DPA; 22:5n-3) and docosahexaenoic acid (DHA; 22:6n-3). | 4 |
| Type of study designs used | A dose-response meta-analysis of prospective and case-control studies. | 4 |
| Study population | Any aged adults (women and men) | 4 |
|  | **Reporting of search strategy should include** |  |
| Qualifications of searchers (e.g., librarians and investigators) | Two trained reviewers (KW and JT) are indicated in the author list. Discrepancies unsolved by discussion during the course of study identification consulted to a third reviewer (DL). | 5 |
| Search strategy, including time period included in the synthesis and keywords | PubMed, EMBASE and Cochrane Library database of systematic review was searched up till February 2014. Search strategy was (“Fatty Acids, omega-3” AND “Colorectal Neoplasms”) for PubMed, (“Colorectal tumor” AND “omega 3 fatty acid”) for EMBASE and (“Fatty Acids, Omega-3” AND “Colorectal Neoplasms”) for Cochrane Library databases. | 5 |
| Databases and registries searched | PubMed, EMBASE and Cochrane Library database of systematic review and meta-analysis. | 4 |
| Search software used, name and version, including special features | We did not employ search software. Endnote was used to merge retrieved citations and eliminate duplications | 6 |
| Use of hand searching | We also searched systematic reviews from the above-mentioned database, and checked reference lists to identify studies that might have been missed. | 5 |
| List of citations located and those excluded, including justifications | The all steps and details of the literature search process are outlined in the flow chart (Figure 1; Table S2). | 5, 6 |
| Method of addressing articles published in languages other than English | Our search was restricted to human studies, and studies published in English. | 5 |
| Method of handling abstracts and unpublished studies | Abstract, unpublished studies and duplicated study were excluded | 5 |
| Description of any contact with authors | We did not contact authors for the detailed information of primary studies and unpublished studies. | 5 |
|  | **Reporting of methods should include** |  |
| Description of relevance or appropriateness of studies assembled for assessing the hypothesis to be tested | Detailed inclusion and exclusion criteria were described in the methods section. | 5 |
| Rationale for the selection and coding of data | Data extracted from each of the studies were relevant to the population characteristics, study design, exposure, outcome, and adjusted confounding factors as covariates. | 5, 6 |
| Assessment of confounding | Restricted the analysis to multiple covariates adjusted estimates. Conducted sensitivity analyses by eliminating studies with possible selection bias. Publication bias was quantitatively examined by Begg’s test and Egger’s regression test. | 6, 7 |
| Assessment of study quality, including blinding of quality assessors; stratification or regression on possible predictors of study results | Subgroup analyses were conducted to identify the sources of heterogeneity by study design, different regions, genders, and tissue types. A random-effects model dose-response meta-analysis was conducted to explore the potential dose-response trend among study populations. | 8 |
| Assessment of heterogeneity | Heterogeneity of the studies were explored within two types of study designs using Cochrane’s Q test of heterogeneity and I^2^ statistic that provides the relative amount of variance of the summary effect due to the between-study heterogeneity. Meta-regression was conducted to compare the difference between subgroups. | 7 |
| Description of statistical methods in sufficient detail to be replicated | Description of methods of meta-analyses for highest exposure quantile compared with lowest, dose-response meta-analysis, subgroup analysis, sensitivity analyses and assessment of publication bias are detailed in the method page. | 7, 8 |
| Provision of appropriate tables and graphics | We provided 2 tables and 6 figures in the manuscript, and 3tables and 7 figures in the supporting in formations. | 8-11 |
|  | **Reporting of results should include** |  |
| Graph summarizing individual study estimates and overall estimate | See meta-analysis results of highest exposure vs. lowest and dose-response | 8, 9 |
| Table giving descriptive information for each study included | See characteristics of the included studies (Table 1) | 8 |
| Results of sensitivity testing | See results of sensitivity analysis and subgroup analysis. | 11 |
| Indication of statistical uncertainty of findings | 95% confidence intervals were presented with all summary estimates, standard mean difference (SMD), I^2^ values, results of sensitivity analyses, and publication analysis. | 10-11 |
|  | **Reporting of discussion should include** |  |
| Quantitative assessment of bias | Q test and I^2^ statistic indicated moderate heterogeneity in strengths of the relationship due to most common biases in observational studies. | 11 & 12 |
| Justification for exclusion | We performed sensitivity analysis omitting individual study to reduce the influence of potential bias on the overall estimate. | 13 |
| Assessment of quality of included studies | We discussed the limitations in our study, and potential reasons for the observed heterogeneity. | 13 |
|  | **Reporting of conclusions should include** |  |
| Consideration of alternative explanations for observed results | There are several hypothesized mechanisms explaining the possible role of LC n-3 PUFA in the etiology of CRC carcinogenesis. | 12 |
| Generalization of the conclusions | Our research findings provided reliable evidence that there was a significant dose-dependent inverse association between biospecimen composition of LC n-3 PUFA and CRC risk, which implied important medical implications. | 14 |
| Guidelines for future research | LC n-3 PUFA as human dietary components may be beneficial in the treatment of CRC. Nevertheless, these conclusions warrants further investigation of prospective cohort studies. | 15 |
| Disclosure of funding source | See acknowledgement | 15 |
