# Supplementary material for: Biospecimen Long-Chain N-3 PUFA and Risk of Colorectal Cancer: A Meta-Analysis of Data from 60,627 Individuals
Source: PLoS One. 2014 Nov 6;9(11):e110574. doi: 10.1371/journal.pone.0110574 (PMC4222788; doi:10.1371/journal.pone.0110574)
Supplement: Table S1 — Observational studies Excluded from Meta-Analysis. (DOC) [file pone.0110574.s002.doc]

**Table S1. Observational studies Excluded from Meta-Analysis**

| **Reference** | **Reasons for exclusion** |
| --- | --- |
| 1. Neoptolemos JP, Husband D, Imray C, Rowley S, Lawson N. Arachidonic acid and docosahexaenoic acid are increased in human colorectal cancer. Gut 1991; 32(3):278-81. | Only comparing LC n-3 composition between normal mucosa and cancer mucosa tissue. |
| 2. Bakker N, Van't Veer P, Zock PL. Adipose fatty acids and cancers of the breast, prostate and colon: an ecological study. EURAMIC Study Group. Int J Cancer 1997; 72(4):587-91. | Only providing average fatty acid composition of adipose tissue aspirates in the Euramic centers. |
| 3. Kobayashi M, Sasaki S, Hamada GS, Tsugane S. Serum n-3 fatty acids, fish consumption and cancer mortality in six Japanese populations in Japan and Brazil. Japanese Journal of Cancer Research 1999; 90(9):914-921. | This is a cross-sectional study. |
| 4. Knutsen SF, Fraser GE, Beeson WL, Lindsted KD, Shavlik DJ. Comparison of adipose tissue fatty acids with dietary fatty acids as measured by 24-hour recall and food frequency questionnaire in black and white adventists: The adventist health study. Annals of Epidemiology 2003;13(2):119-127.  . | Evaluating correlations between proportion of individual fatty acids (% of total fat) in 24-hour recalls and FFQ vs. adipose tissue. |
| 5. West NJ, Clark SK, Phillips RK, et al. Eicosapentaenoic acid reduces rectal polyp number and size in familial adenomatous polyposis. Gut 2010;59(7):918-25. | This is a RCT study. |
| 6. Szachowicz-Petelska B, Sulkowski S, Figaszewski ZA. Altered membrane free unsaturated fatty acid composition in human colorectal cancer tissue. Mol Cell Biochem 2007;294(1-2):237-42. | Evaluating content of free unsaturated fatty acids in colorectal adenocarcinoma and normal colon mucosa. |
| 7. Kondo Y, Nishiumi S, Shinohara M, et al. Serum fatty acid profiling of colorectal cancer by gas chromatography/mass spectrometry. Biomarkers in Medicine 2011;5(4):451-460. | Comparison of fatty acid levels between the colorectal cancer patients and healthy volunteers according to clinical stage. |
